# Estimating the spatial position of marine mammals based on digital camera recordings

**DOI:** 10.1002/ece3.1353

**Published:** 2015-01-08

**Authors:** Jeroen P A Hoekendijk, Jurre de Vries, Krissy van der Bolt, Jens Greinert, Sophie Brasseur, Kees C J Camphuysen, Geert Aarts

**Affiliations:** 1 IMARES Wageningen URDen Burg, the Netherlands; 2 Department of Marine Evolution and Conservation, University of GroningenGroningen, the Netherlands; 3 Royal Netherlands Institute for Sea Research (NIOZ)Den Burg, the Netherlands; 4 Department of Ecology, Utrecht UniversityUtrecht, the Netherlands; 5 GEOMAR Helmholtz Centre For Ocean Research KielKiel, Germany; 6 Department of Aquatic Ecology & Water Quality Management, Wageningen UniversityWageningen, the Netherlands

**Keywords:** Distance, fine-scale distribution patterns, harbour porpoise, marine mammal, photogrammetric, theodolite, tide, video camera

## Abstract

Estimating the spatial position of organisms is essential to quantify interactions between the organism and the characteristics of its surroundings, for example, predator–prey interactions, habitat selection, and social associations. Because marine mammals spend most of their time under water and may appear at the surface only briefly, determining their exact geographic location can be challenging. Here, we developed a photogrammetric method to accurately estimate the spatial position of marine mammals or birds at the sea surface. Digital recordings containing landscape features with known geographic coordinates can be used to estimate the distance and bearing of each sighting relative to the observation point. The method can correct for frame rotation, estimates pixel size based on the reference points, and can be applied to scenarios with and without a visible horizon. A set of R functions was written to process the images and obtain accurate geographic coordinates for each sighting. The method is applied to estimate the spatiotemporal fine-scale distribution of harbour porpoises in a tidal inlet. Video recordings of harbour porpoises were made from land, using a standard digital single-lens reflex (DSLR) camera, positioned at a height of 9.59 m above mean sea level. Porpoises were detected up to a distance of ∽3136 m (mean 596 m), with a mean location error of 12 m. The method presented here allows for multiple detections of different individuals within a single video frame and for tracking movements of individuals based on repeated sightings. In comparison with traditional methods, this method only requires a digital camera to provide accurate location estimates. It especially has great potential in regions with ample data on local (a)biotic conditions, to help resolve functional mechanisms underlying habitat selection and other behaviors in marine mammals in coastal areas.

## Introduction

As marine mammals spend most of their time under water, it is challenging to study spatial and temporal patterns in their distribution relative to topographic and oceanographic conditions, or conspecifics. The small and elusive harbour porpoise (*Phocoena phocoena*) is particularly hard to observe in the wild. However, all marine mammals appear at the surface to breath, and location estimates of these surfacing events can be used to link distribution patterns with habitat characteristics. The objective of this study was to develop a method to make precise location estimates of surfacing marine mammals, with or without a visible horizon, using video recordings.

Video recordings of harbour porpoises were made from a relatively low land-based observation platform, using a standard digital single-lens reflex (DSLR) camera, in a sea strait with ample data on local environmental conditions. These platforms have the advantage of being nonintrusive, and the behavior of the studied animals is not disturbed. Most studies on the distribution of porpoises are based on aerial or ship-based surveys (e.g., Hammond et al. 2002; Embling et al. 2010; Gilles et al. 2011; Scheidat et al. 2012). Such moving platforms normally provide only a single snapshot of individuals in a highly dynamic marine environment, where tidal conditions (e.g., stratification and frontal systems) may change on a timescale of hours (e.g., de Vries et al. 2014).

Earlier land-based cetacean surveys have often used theodolites (Cox et al. 2001; Culik et al. 2001; Koschinski et al. 2006; Sagnol et al. 2014), but these can be impractical for studies of harbour porpoises. Theodolites have to be pointed exactly at the sighted individual in order to take readings, while porpoises are normally only briefly visible at the surface, making three to four highly inconspicuous rolling movements. A further limitation of theodolite studies is that it is impossible to record more than one animal simultaneously, resulting in a loss of information, such as spatial group structure. When using video recordings, the playback feature assures that the geographic position of groups of porpoises can be recorded in detail.

In this photogrammetric approach, the recordings are used to make angular measurements of the porpoise (or indeed any other object at the sea surface) relative to the horizon or a known shoreline (Lerczak and Hobbs 1998; Gordon 2001; Leaper and Gordon 2001). The vertical angle between object and the horizon (or known shoreline) is used to calculate the distance between the camera and the object. The bearing of the sighting can be calculated by measuring the horizontal angle between the object and a reference point with known coordinates. When both the distance and bearing of the sighting are known, the exact geographic position of the object can be calculated.

A similar technique was used by Denardo et al. (2001) while studying interanimal distance in pods of killer whales (*Orcinus orca*): They used a theodolite to determine the location of a reference animal and used video recordings to determine the position of other pod members relative to this reference animal. Hastie et al. (2003) further developed this method by making angular measurements of surfacing bottlenose dolphins (*Tursiops truncatus*) relative to a metal wire frame erected in front of the camera to examine spatial distribution patterns. Here, we show how landscape features can be used as a reference instead, to estimate fine-scale spatiotemporal locations of surfacing harbour porpoises.

We first describe the mathematical equations underlying the photogrammetric techniques and facilitate the use of this method by developing a publically available script for R (http://www.R-project.org/) that calculates the exact geographic position of porpoises based on video recordings. The accuracy of the method presented is tested in two calibration experiments. Finally, we present examples illustrating how information on the spatiotemporal distribution of surface events of harbour porpoises could potentially be used in relation to high-resolution information on tidal currents and bathymetry. This study focuses on the occurrence and the spatial distribution of harbour porpoises within a large sea strait.

## Materials and Methods

### Research area

**Figure 1:**
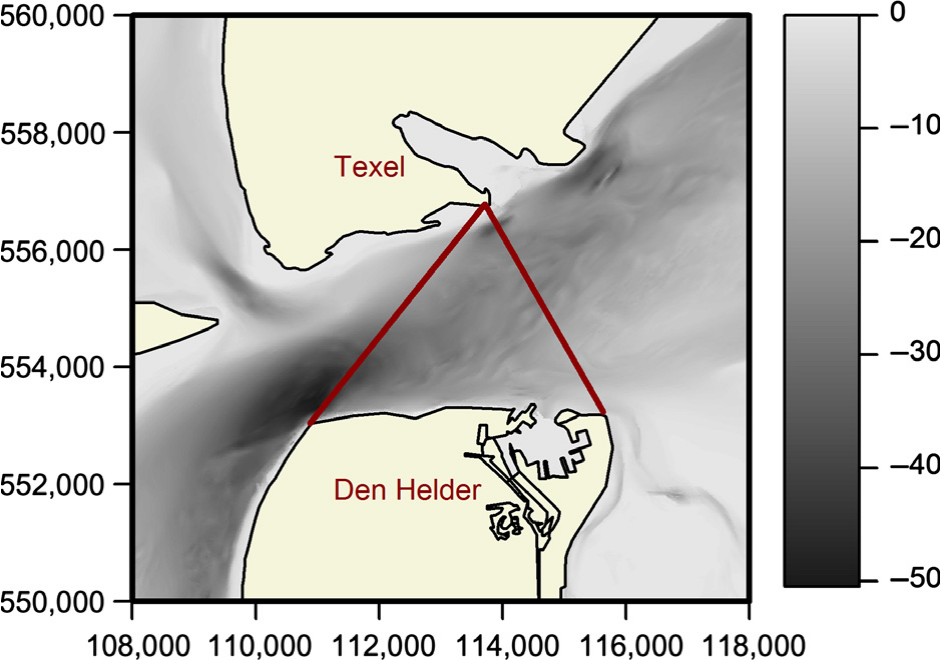
The Marsdiep inlet plotted with available bathymetric data. Boundaries of the research area are plotted as red lines. Coordinates are in the Dutch coordinate system (“Rijksdriehoekscoördinaten”).

The study was carried out in the Marsdiep inlet, a tidal inlet connecting the North Sea and the western Dutch Wadden Sea between the island of Texel and the city of Den Helder on the Dutch mainland (Fig.1). A pilot study by Boonstra et al. (2013) showed that many porpoises were sighted in the northeastern part of the Marsdiep inlet (Fig.1) in late winter and early spring. The oceanographic characteristics of the inlet are well studied (Cadee and Hegeman 1979; Ridderinkhof et al. 2002; Merckelbach and Ridderinkhof 2006; Buijsman and Ridderinkhof 2007) and are characterized by large hydrographic and bathymetric variability, which makes it ideal for investigating fine-scale habitat selection (Albert et al. 2010). The mean depth of the area is 23 m (max 37 m), but varies strongly on a small spatial scale. Land-based observations were made from Texel (52˚59′47N, 4˚46′20E; Fig.1). Characteristic landmarks of Den Helder, including apartment blocks, churches, and a light house, are clearly visible from the observation post at Texel (Fig.1, ∽3.5 km distance).

### Field protocol

Visual observations were conducted between 8:00 and 17:30 CET, on days between January 24 and April 11, 2012, and between February 26 and April 21, 2013. The observation schedule was set up to obtain at least two scans for every hour, throughout this period. Environmental conditions (i.e., precipitation, sea state, glare, cloud coverage, and estimated viewing distance) were scored every 15 min. In search for harbour porpoises, the area was scanned continuously by the naked eye. Every ∽10 min, a scan of the entire area was made with binoculars (Swarovski 10 × 42 EL). The binoculars were aligned with a digital single-lens reflex (DSLR) Canon (Melville, NY) 600d camera with a Sigma (Ronkonkoma, NY) 70–300 mm F4-5.6 DG OS lens, which was mounted on a tripod. The entire field of view of the binoculars was thus recorded. As soon as a harbour porpoise was detected, a camera recording was started and the porpoise was followed using the binoculars. When multiple animals were detected, one focal animal was chosen, but individuals in the direct vicinity were also recorded. The internal clock of the camera was set at Central European time (CET). For each recording, the end time and duration were automatically stored in the details of the video files. The camera height was 9.59 m (±3 cm) above mean sea level (NAP, Amsterdam Ordnance Datum), measured with a differential global positioning system (Trimble, Sunnyvale, CA) R4 DGPS and d Nomad 900 GXC cellular modem). Spoken comments were recorded on the audio channel to support the detection of porpoises during digital analysis.

### Mathematical determination of porpoise locations

When the exact geographic position and height of the camera is known, only the distance to a surfacing porpoise and the bearing of the sighting are required to calculate its geographic position. To calculate the distance between the porpoise and the camera, the vertical angle between the porpoise and a reference point is required, for which often the horizon is used (Gordon 2001). The bearing of the sighting can be calculated by measuring the horizontal angle between the porpoise and a recognizable feature with known geographic coordinates (i.e., reference point), visible in the image. This reference point can be any feature in the landscape (e.g., building, rock, etc.) or an object placed artificially in the field of view. In Appendix S2, we show how to calculate the coordinates of a sighting using the horizon and a single reference point.

Under certain conditions, the horizon might not be visible, for instance, in estuaries or tidal inlets. The distance and bearing of the sighting can then be calculated without using the horizon, using two reference points instead of one, both have to be located at the sea surface in the approach presented here. An advantage of using two reference points is that the pixel size (in radians) can be derived from the image directly, so that the focal length of the lens during the recordings does not need to be known. Therefore, focal length can change between recordings, for example, to zoom in or out depending on the location of the animals. Because in our setup, the horizon was not visible, possible sidewards tilting of the camera may not be apparent. To correct for this, an artificial horizontal line needs to be constructed through one of the reference points, which requires rather extensive calculations (see step four below and Appendix S1).

#### 1 The interior spherical angles

**Figure 2:**
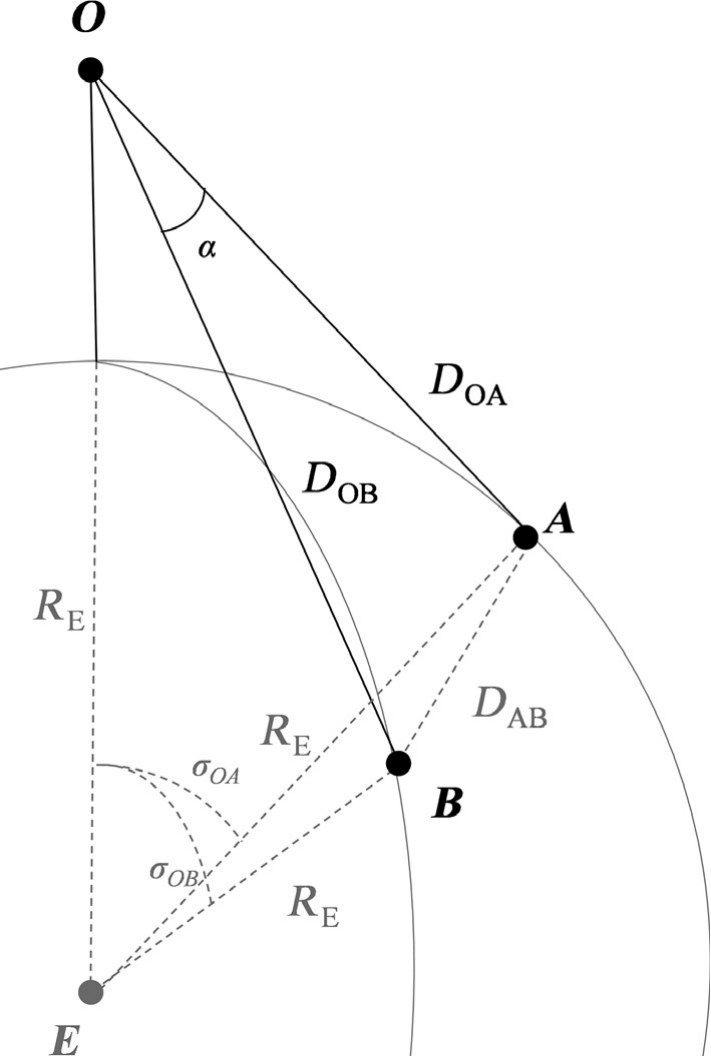
Schematic 3D view of Earth with the position of the observer (*O*) and reference points (*A* and *B*), where *R*_*E*_ is the Earth's radius, *D*_*OA*_ and *D*_*OB*_ are the direct distances between *O* and reference *A* and *B,* respectively (eq. 2), *D*_*AB*_ is the direct distance between point *A* and *B*. *σ*_*OA*_ and *σ*_*OB*_ are the interior spherical angles between *O* and reference *A* and *B,* respectively (eq. 1), and *α* is the angle between *A*,*O,* and *B* (eq. 4).



First, the interior spherical angle or central arc angle (*σ*), between the observer (*O*), the center of the Earth (*E*), and a first reference point (*A*) was calculated using their geographic coordinates (see Fig.2)

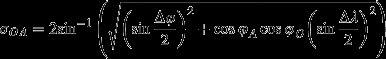
1

where *φ*_*A*_ is the latitude of *A*,*φ*_*O*_ is the latitude of *O*, Δ*φ* is the difference in latitude between *A* and *O*, and Δ*λ* is the difference in longitude between *A* and *O*. The same equation was used to calculate the interior spherical angle (*σ*_*OB*_) between the observer and a second reference point (*B*), and between the two reference points (*σ*_*AB*_).

#### 2 The angle between the first reference point, the observer, and the second reference point

The horizontal angle (*α*) between the two reference points and the observer can be calculated based on the straight-line distances between the observer and reference point *A* (*D*_*OA*_), the observer and reference point *B* (*D*_*OB*_), and between reference point *A* and *B* (*D*_*AB*_) (see Fig.2).

Using the law of cosines, the straight-line distance between *A* and *B* (*D*_*AB*_) was calculated based on the mean radius of the Earth (*R*_*E*_ = 6371008 m (Moritz 1992)), and the interior spherical angle between *A* and *B* (*σ*_*AB*_, eq. 1)

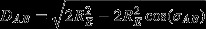
2

Similarly, the straight-line distances between the observer and reference point *A* (*D*_*OA*_) are defined as

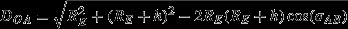
3

where *h* is observer height. This equation was also used to calculate the distance between the observer and the second reference point *B* (*D*_*OB*_). The angle between the two reference points from the observer point of view (*α*, see Fig. A1 in Appendix S1) was calculated, again using the law of cosines

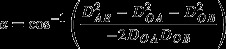
4

#### 3 Individual pixel size (in radians)

When pixels are square, the Pythagorean theorem can be used to calculate the distance (in pixels) between reference point *A* and *B* (*L*_*AB*_) using the pixel coordinates of the two reference points (*A*_*y*_*A*_*x*_ and *B*_*y*_*B*_*x*_) in the video frame.

**Figure 3:**
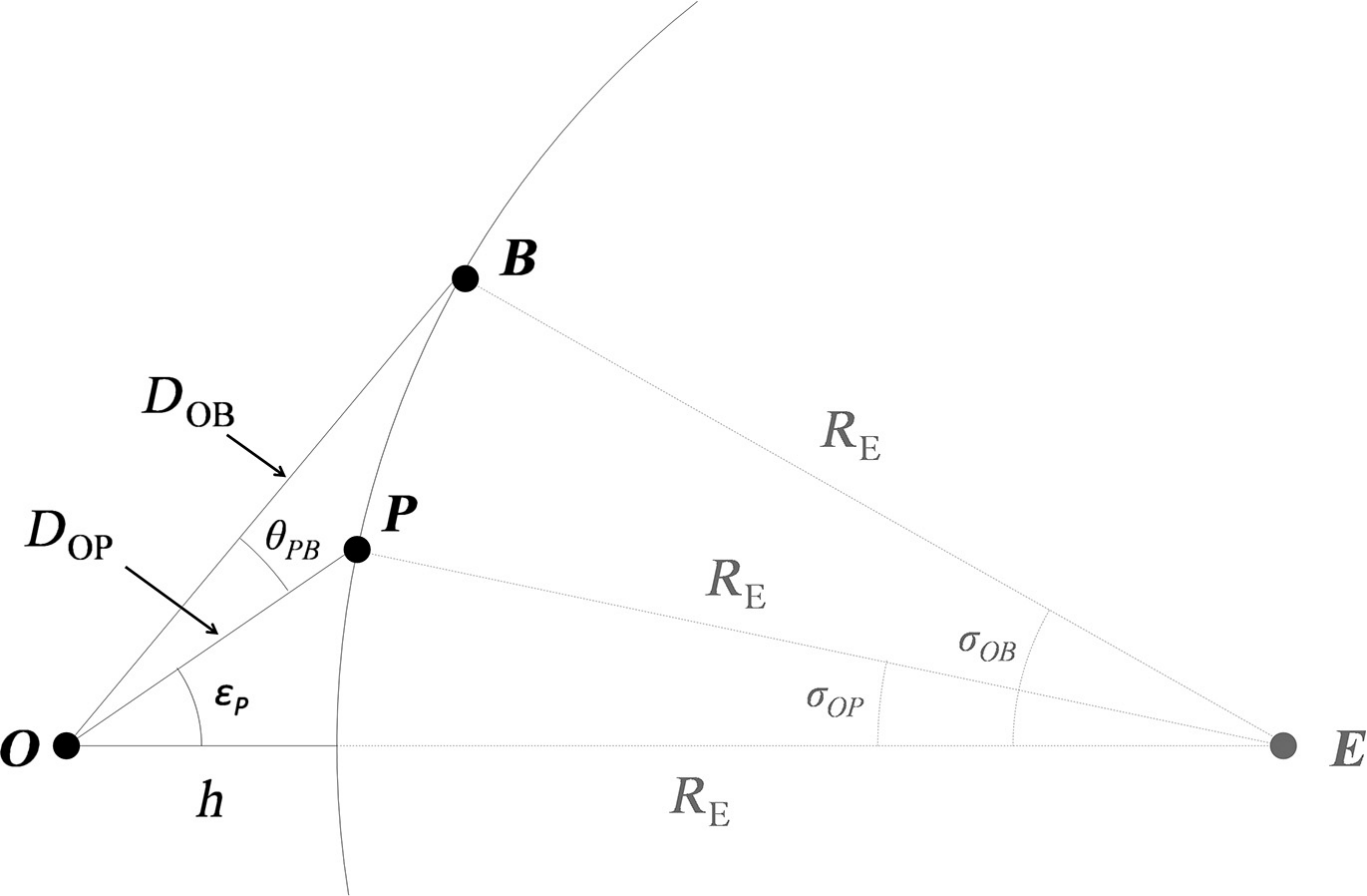
Schematic 2D cross section of Earth with the position of the observer (*O*), reference point *B,* and the observed porpoise (*P*). Because point *P’* is the projection of *P* onto the horizontal line through *B*, point *P’* is also located on point *B* in this Figure. *E* is the center of the Earth, *R*_*E*_ is the Earth's radius, *D*_*OP*_ and *D*_*OB*_ are the direct distance between *O* and *B* and between *O* and *P* respectively (eqs 3 and 15), *σ*_*OP*_ and *σ*_*OB*_ are the interior spherical angles between *O* and *P* and between *O* and *B,* respectively (eqs 1 and 16), *ε*_*A*_ is the vertical angle between *A*,*O,* and *E* (eq. 7), and *θ*_*PB*_ is the vertical angle *P'OP* (eq. 13a).



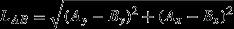
5

The size of an individual pixel (*q*, in radians) can now be defined as

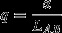
6

#### 4 The horizontal line through reference point B

To calculate the distance between the observer and porpoise (*P*), it is necessary to estimate the vertical angle between the porpoise perpendicular to an artificial horizontal line, which can be constructed from two reference points. The vertical angle (*ε*_*A*_) between *A*,*O,* and the center of the Earth (*E*) was determined using *D*_*OA*_ (see eq. 3) and by applying the law of cosines

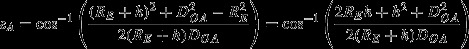
7

This same equation was also applied to reference point *B* (i.e., *ε*_*B*_). Appendix S1 demonstrates how to derive the slope of the artificial horizontal line in the frame through *B* (*m*_*BC*_). Using the pixel coordinates of point B in the image (*B*_*y*_ and *B*_*x*_), the intercept (*c*_*BC*_) of the artificial horizontal line through *B* is

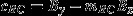
8

#### 5 The horizontal and vertical angle between reference point B and the porpoise

Line *PP′* is shortest distance between the porpoise (*P*) and the horizontal line *CB*, hence the slope (*m*_*PP’*_) is

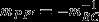
9

and similar to eq. 8, the intercept (*c*_*PD*_) of line *PP′* through *P* (with pixel coordinates *P*_*x*_ and *P*_*y*_) can be determined with

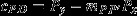
10

The coordinates of point *P′* are located at the intersection between line *PP′* and the horizontal through *B* and can be calculated as follows:

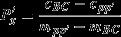
11a

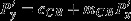
11b

Now that the coordinates of point *P′* are known, the length of line *BP′* and line *PP′* can be calculated with

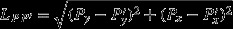
12a

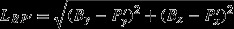
12b

This entire calculation (eqs 8-12) may appear rather cumbersome, but is required to correct for any misalignment of the camera. Perfect alignment of the camera results in *L*_*PP′*_ being simply the difference between *B*_*y*_ and *P*_*y*_ and *L*_*BP′*_ being the difference between *B*_*x*_ and *P*_*x*_.

*L*_*PP′*_ and *L*_*BP′*_ are in pixels, but can be transformed to radians by multiplication with the individual pixel size (*q*). This results in the vertical angle *P'OP* (*θ*_*PB*_) and horizontal angle *P'OB* (*γ*_*PB*_) between the porpoise *P* and reference point *B,* respectively.

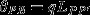
13a

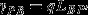
13b

#### 6 The interior spherical angle between porpoise and observer

The vertical angle (*ε*_*P*_) between the porpoise (*P*), the observer (*O*), and the center of the Earth (*E*) is defined as

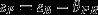
17

where *ε*_*B*_ is the vertical angle *EOB* (eq. 7, Fig.3). Using the law of cosines, the straight-line distance between the observer and the harbour porpoise (*D*_*OP*_) is

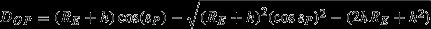
15

Finally, the interior spherical angle between the observer and the porpoise (*σ*_*OP*_) was calculated using the law of sines.

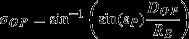
16

#### 7 The porpoise geographic position

Now that the interior spherical angle from the observer relative to the harbour porpoise (*σ*_*OP*_) and the bearing of the sighting relative to reference point *B* (*γ*_*PB*_) are known, the exact location of the porpoise can be calculated as well. First, the bearing (*κ*) from the observer to the porpoise was determined by calculating the bearing between the observer and reference point *B*, and adding *γ*_*PB*_.

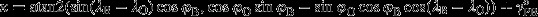
17

Because the latitude and longitude coordinates are defined in degrees, all parameters in eq. 17 are in degrees, and so is 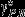. The function atan2 is defined as

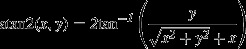
18

The latitude of the porpoise was calculated using

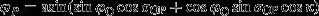
19

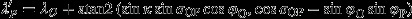
20

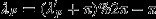
21

where *λ*_*O*_, *φ*_*O*_, *λ*_*P*_, and *φ*_*P*_ are longitude and latitude coordinates of the observer and porpoise, respectively. % is the modulo. These equations (eqs 17-21) are derived from the functions “bearing” and “destPoint” of the R geosphere-package (Hijmans et al. 2012).

## Image analysis

**Figure 4:**
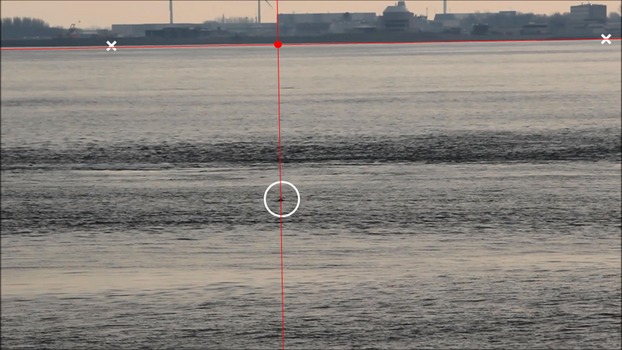
Screenshot of the R script (Data S1). Reference points (white crosses) are located on the waterline directly underneath landmarks on the opposite side of the Marsdiep. The distance between the porpoise (circle) and the horizontal (constructed through the right reference point) was determined to calculate the vertical angle. The distance between the intersection (red dot) and the right reference point was used to calculate the horizontal angle.

The video footage was displayed with the program Imagegrab (http://paul.glagla.free.fr/imagegrab_en.htm). Spoken comments on all sightings of porpoises were recorded on the audio channel of the video camera. In most cases, this cue was used to aid in the detection of porpoise on the screen. When a porpoise was detected, the video recording was rewinded frame-by-frame, back to the moment where the dorsal fin of the porpoise was at its highest point. This frame was saved as a.jpeg. The name of the original movie file, as well as the time and frame number, was stored within the file name. After completion, the frames were loaded into R (R-Development-Core-Team 2011) as well as the following data: 1) sighting details including an id code for individual recognizable porpoises, swimming direction, and starting time of the original movie file; 2) sea surface elevation data at Den Helder (52°57.86′N, 4°44.70′E; www.waterbase.nl) used to recalculate observation height relative to the sea surface; and 3) a list of reference points situated at the sea surface, with their corresponding GPS coordinates. The reference points of this study were located on the opposite shoreline of Den Helder, directly underneath characteristic buildings. Next, each frame was plotted, and the coordinates (in pixels) of the two reference points and the surfacing porpoise in the picture were determined using the R-function “locator” (Fig.4). The pixel size (eq. 6) was estimated for each frame containing at least two reference points. The pixel size of all frames within a single recording was averaged and used for further calculations. Using this approach, the focal distance of the lens can be adjusted between recordings.

## Accuracy testing and sensitivity analysis

To test the accuracy of the location estimates, two calibration experiments were conducted. First, video recordings of a canoe equipped with a handheld Garmin 60csx GPS were made. Similar to a porpoise sighting (see above), a series of 37 frames was extracted, and the location of the canoe was determined using the video technique and compared with the actual GPS measurements. This technique allows for an estimate of the error at different locations (and distances), using different reference points, within a relative short time window of 30 min, during which there was little variability in tidal height. Second, for the data collected in both 2012 and 2013, 38 frames containing a navigational measurement pole with known location (52.99400° N, 4.77205° E) were used to estimate the geographic location of that pole. The pole was located at a distance of 266 m from the observer. Frames were selected throughout one tidal cycle, and the observed error should reflect the effect of unaccounted variation in tidal height compared to the height reported in www.waterbase.nl, which was used in the calculations. For each location estimate, the error parallel and perpendicular to the bearing, as well as the mean error (in m) was estimated, the latter being defined as the absolute distance between the GPS location of the pole/canoe and estimated location.

The largest error in the location estimate is most likely caused by incorrect estimation of the camera height, for example, due to imprecise tidal height measurement. To investigate the effect of such height miss-specifications, we added, respectively, 1, 10, and 100 cm to the camera height (and reduced the radius of the earth with an equal amount) and re-estimated the canoe locations. The additional error was shown as function of the distance between the location of the canoe and camera observation point.

## Example applications

For the estimated geographic positions of the porpoises, a number of different applications were explored. Distributions patterns were plotted over time and for different tidal states and overlaid with high-resolution (1 by 1 m) multibeam bathymetry map. Multibeam data were collected by the Ministry of Infrastructure and the Environment (“Rijkswaterstaat”) in January 2012 with an EM3002 multibeam (300 kHz, 1.5 by 1.5 beam angle). Data were presented fully processed (motion compensated and sound velocity corrected) as xyz coordinates. Data were visualized using R (R-Development-Core-Team 2011).

Also, we examined movement patterns, travel speeds, and dive duration of individual animals. The objective of these applications was not to provide detailed biological insight into the behavior and ecology of the harbour porpoise, but to demonstrate the accuracy and applicability of the porpoise-location method.

## Results

### Geographic location estimates and errors

**Figure 5:**
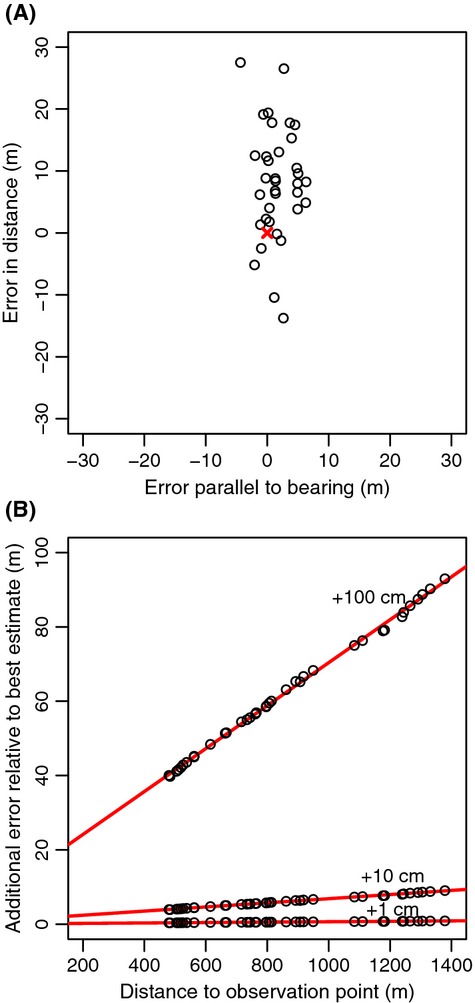
Error in the location estimate of the canoe calibration experiment. (A) Error in the location estimate perpendicular to the bearing (i.e., error in the distance) plotted against the error parallel to the bearing. Red cross marks the canoe GPS location centered at 0. (B) Additional error in the location estimates caused by miss-specifications of the camera height. Here, 1, 10, and 100 cm were added to our estimated camera height.

To determine the accuracy of the estimated sighting locations, we first compared the true GPS measurements of the canoe with the calculated positions derived from the video analysis. The distance between the camera and the canoe ranged from 480 to 1378 m. The difference between the GPS measurements and the calculations was on average 12.0 m (SD = 8.8), which consisted of two sources of errors, namely the error in the estimation of the distance (11.4 m, SD = 9.0) and the error in the bearing (2.4 m, SD = 1.92), see Fig.5a. It should be noted that the (undefined) error in the GPS location estimate of the canoe might also be several meters, and therefore, the actual error might be different. The error in the estimate of the distance to the camera was mostly the result of a bias of 9.0 m (Fig.5a). We expect this to be the result of inaccurate measurements of the tidal height and therefore also the height of the camera. The sensitivity analysis indeed shows that an error in the height of only 10 cm can lead to a bias of 9 m at the maximum distance of 1400 m (Fig.5b).

**Figure 6:**
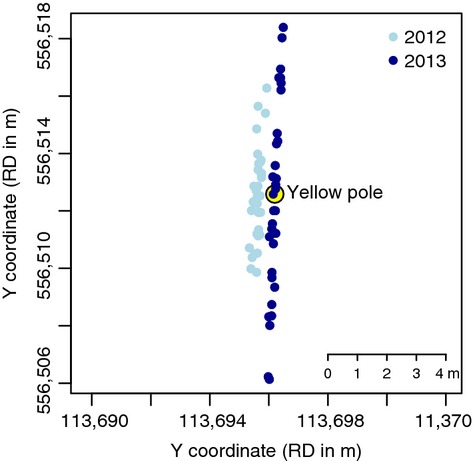
Geographic locations of pole in the Marsdiep as estimated by the camera technique compared to the actual location.

Secondly, the comparison between the estimated location of the pole and the actual location showed that the mean error was 2.35 m (SD = 1.73). Almost all errors were along the line between the observer and the pole (Fig.6), illustrating that the error in the estimated bearing is much smaller than the error in the distance estimate. There was an eastward shift of approximately 1 m apparent between 2012 and 2013, which is most likely the result of the pole (positioned within the sand) to have shifted during that year.

To illustrate the potential of the method to estimate the distribution of porpoise sightings, video recordings of 62 days in 2012 and 2013 were analyzed, corresponding to 274 h of survey time. Observation effort was 138 h during ebb and 137 h during flood tide.

A total of 3165 porpoise sightings were obtained, of which 1669 before high water and 1496 after high water. Porpoises were observed at a mean distance of 596 m (min 64 m, max 3136 m). 13.3% of the sightings were followed within the same recording by a second sighting (of the same or another individual) within 3 s, and 41.1% of the sightings had a consecutive sighting within 6 s.

**Figure 7:**
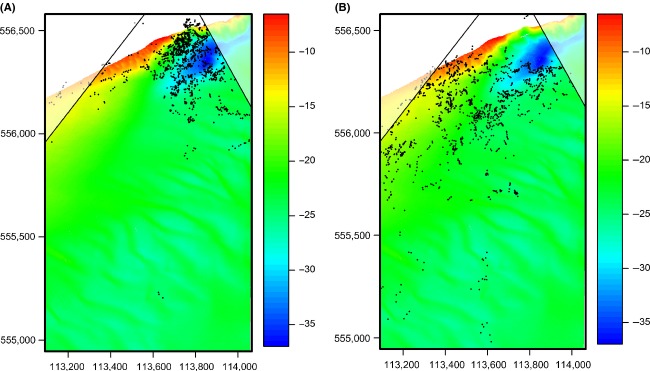
Porpoise sightings plotted against water depth (in m, data from RWS), during flood (period between the low and high water peak) (left) and ebb (right). Approximately 1–2 h before high and low water, the flood currents are at its strongest and run in NNE and ESE direction, respectively. The nonshaded region defines the area in which existing reference points were located.

The estimated sighting locations are shown in Fig.7, along with water depth. During ebb tide, most sightings were located in the shallow western part of the study area. During flood, sightings were concentrated around the deepest part of the research area. The largest aggregations of porpoise sightings in this area occur during flood and appear to be concentrated along the edge of a deep hole in the seabed (Fig.7). Porpoise density was particularly high along the northern edge of the hole, where the slope of the seabed is steepest (distance from observer ∽360 m), while in the deepest part of the hole (distance ∽450 m), there were very few sightings. The number of sightings increased again along the southern slope (distance ∽550 m). Sighting rates near the hole in the seabed were highest 2 h before the high water peak. During this late flood phase, when the magnitude of the flood current decreases, strong cross-stream currents are often observed in the Marsdiep basin (de Vries et al. 2014).

### Behavioral observations

**Figure 8:**
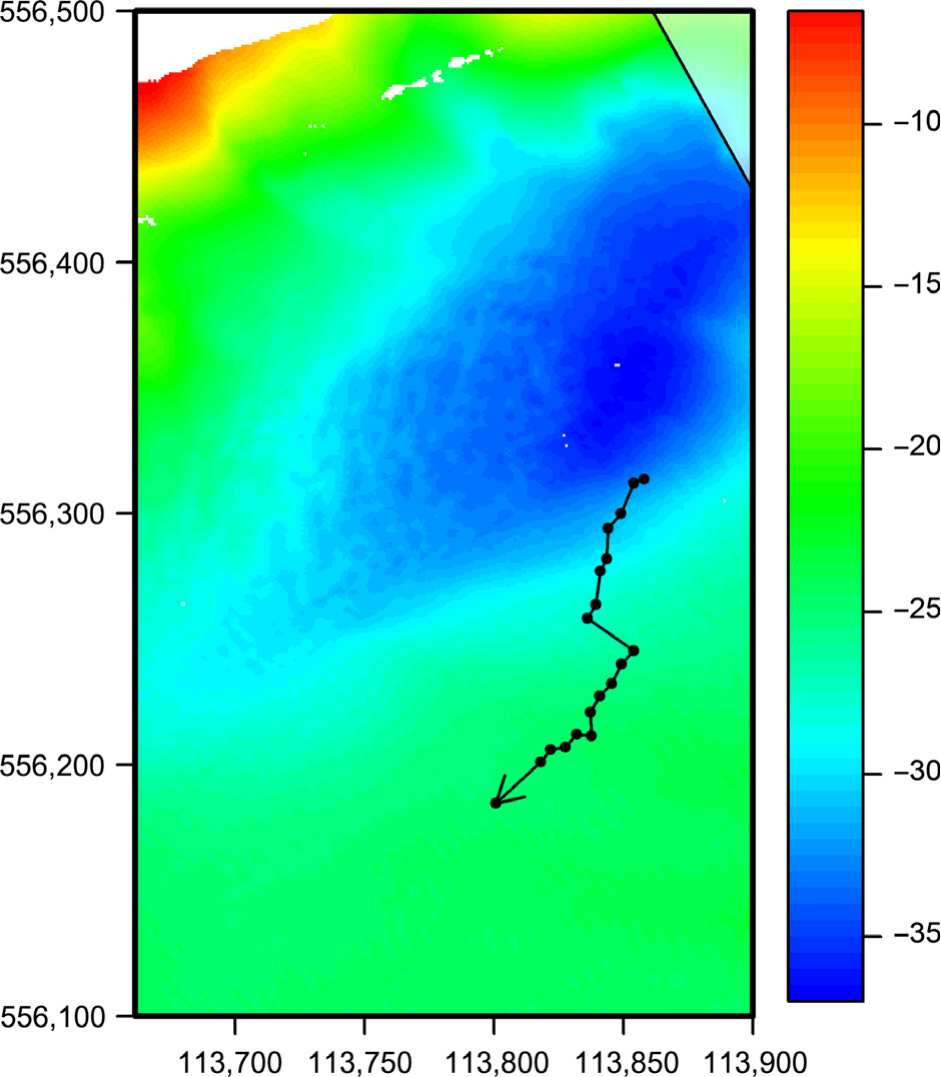
Movement of a single porpoise moving in the same direction as the current (W/SW) with an average speed of 0.58 m/s. For more details, see Fig.6. The porpoise was tracked for 7 min, on March 3, 2012, from 9:21 up to 9:28, over a distance of 172 m.

The use of digital cameras enables the collection of detailed information on location, time, and body orientation, which can be used to derive behavioral characteristics of the animal such as swimming direction, dive duration, and travel speed. Travel speed is defined as the speed relative to the sea floor and differs from swimming speed because no correction for the current velocity is made. However, if independent current measurements are available, actual swimming speed could be estimated. Fig.8 shows the movement of, most likely, one individual, recorded 4 h after the high water peak, during ebb tide. A series of shorter dives (average duration 13.8 s) was followed by a longer dive (duration 85.0 s). Travel speed was on average 0.58 m/s, and the animal was moving in the same direction as the main current.

## Discussion

### Estimating fine-scale spatiotemporal distribution

The objective of this study was to illustrate how a regular DSLR camera can be used to estimate the spatiotemporal fine-scale distribution of marine mammals at sea and to provide the software to carry out the estimation. While observations were made from a height of only 9.59 m, we were able to detect porpoises up to a distance of up to 3136 m (mean distance 596 m) and estimate their location with an estimated mean error of less than 12.0 m. In total, 41% of the sightings were followed by a consecutive sighting within 6 s, and several individuals surfaced almost simultaneously. Such rapid succeeding observations would have been difficult to record using traditional theodolites.

The photogrammetric method presented here only requires a camera, and as long as a reference point with known coordinates is visible in the frame, it can be used to estimate the geographic position of any animal or object at the sea surface. For example, another possible implementation is to estimate the size of objects floating at the sea surface, such as oil slicks, visible boundaries between different water masses or body size estimates (Lacey et al. 2009).

### Sources of error in the location estimates

Errors in several parameters could lead to inaccurate marine mammal location estimates. Which of those errors will be most influential will be study specific. Here, the observation platform was relatively low compared to the distance at which porpoises were detected. Therefore, particularly errors in the estimation of the height can lead to substantial errors in the estimation of the distance to the sighting. This is shown in Fig.5, where an error of 10 cm can lead to an error of approximately 7 m of sightings 1 km away. The error in the camera height could be due to the inaccuracy of the DGPS height estimate; however, as the latter error is only a few centimeters, its effect on the location estimate will be minor. However, the inaccuracy of the water level is probably the largest source of error. Tidal data were collected at Den Helder, which is approximately 3 km from observation point. A comparison with some existing tidal data collected 1 km east of the observation point shows that the difference in tidal height measured in Den Helder could be as large as 20 cm. Other sources of error are inaccurate estimation of the location of reference points. When reference points are used that are located at great distance from the observer (like in this study), this source of error is expected to be very minor. However, when the reference points are close to the camera (e.g., artificial reference points several meters from the camera), small errors in their location estimates will have a relatively large effect on the angular measurements.

### Strengths and limitations

The main advantages of the method presented in this study are that it is relatively cheap, easy to employ, and takes little preparation time. Despite the simplicity, it results in accurate location estimates with a relative small error (12.0 m), for sightings up to 1 km. When animals are sighted at larger distances (several kms), the error increases and it might also be necessary to correct for atmospheric refraction of light (Leaper and Gordon 2001). However, our approach is less susceptible for refraction than a method that uses the horizon, because the distance between the observer and the reference points is smaller.

For the location estimates presented here, additional information, such as the exact geographic location and height of the camera, the geographic location of reference points, and water level are required. These can be determined at any time, for example, after a sufficient amount of marine mammal sightings are recorded. This can be beneficial when investigating the spatial distribution of marine mammals, which can be unpredictable in their occurrence. Initial sampling effort could be distributed over different areas, and based on the available data of a region, this extra information can be collected. Finally, this camera system allows for continuous recordings of individuals, even when they appear at the surface only very briefly (e.g., the harbour porpoise), and for repeated sightings of the same individuals or simultaneous recordings of different individuals. If the camera is levelled perfectly horizontally, the calculations can be simplified and only a single reference point is required. In other scenarios, the horizon can be used to correct for misalignment of the camera and to calculate the distance to the object, while the reference point is used only to calculate the bearing of the sighting (Appendix S2).

In the absence of reference points, artificially placed objects (e.g., poles) with known position in the view of the camera can be used. In this situation, pixel size needs to be calculated separately by a calibration experiment. Although this study was mainly focused on estimating the location based on two reference points, the R-script to estimate the location using only a single reference point is provided as well (see Supporting Information).

### Ecological applications

For impact assessment, mitigation and species conservation, it is necessary to understand spatial distribution patterns of marine mammals and why they utilize or prefer certain regions over others. Understanding which habitat types are used or preferred by porpoises and under which conditions, not only requires detailed information on porpoise distribution, but also on the local physical and biological conditions. The Marsdiep area is a very suitable study area, as data on the fine-scale distribution of porpoises can be collected in a marine system with ongoing environmental monitoring of (a)biotic variables. This study demonstrated that aggregations of porpoises occurred in an area dominated by a deep hole and steep gradients in the seabed, possibly corresponding to strong lateral gradients in current velocity, or to the presence of strong cross-stream currents. Sighting rates were highest 1–2 h before the high water peak, during the late flood phase, when cross-stream currents are strongest in the Marsdiep basin (de Vries et al. 2014).

The calculated geographic positions can also be compared with other variables. A spatial survey of currents, salinity, and temperature can be used to link porpoise sightings to fronts or upwelling regions. Linking sightings with local current estimates also allows for estimations of swimming speed. Porpoise positions may also be compared with biotic variables. Local prey abundance and distribution could be sampled in areas with high sighting rates, preferably during the same period when recordings of porpoises are made. Ultimately, understanding the mechanisms underlying the selection of fine-scale topographic and hydrodynamic features could help to understand the distribution of porpoises for other areas.

Most studies on the distribution of harbour porpoise collect single snapshots on relatively large spatial scales using aerial or ship-based surveys (Embling et al. 2010; Gilles et al. 2011; Scheidat et al. 2012). Recently, porpoises have been equipped with satellite relay data loggers which allows for the remote recording of individual movements at different spatial scales (Sveegaard et al. 2011). However, in addition to potential financial and ethical considerations, there is little control on where distribution data are collected, and it may occur in regions without spatiotemporal high-resolution environmental data.

As anthropogenic use of the marine environment continues to increase, it is important to come to a more mechanistic understanding of porpoise habitat preferences and how animals respond to natural and anthropogenic changes. For instance, while an increase in porpoise occurrence was observed in a Dutch offshore wind farm (Scheidat et al. 2011), the opposite was observed in Denmark (Carstensen et al. 2006). In order to estimate the impact of wind farms on porpoises, data on their fine-scale distribution relative to the individual windmills and the boundaries of the park are needed. Clearly, the scale of study is important (Pribil and Picman 1997; Hastie et al. 2003). This method allows for fine-scale, nonintrusive estimates of the spatiotemporal distribution patterns. When combined with more detailed information on (a)biotic variables, it may ultimately allow for a better understanding of the functional mechanism behind habitat selection in harbour porpoises.

## Supporting Information

**Appendix S1.** Correcting for picture frame rotation. **Appendix S2.** Estimating the spatial position based on the horizon and a single reference point.

**Data S1.** Description of procedure to define porpoise location.

**Data S2.** R-code to calculate position of harbour porpoises without horizon.

**Data S3.** R-code to calculate position of harbour porpoise with horizon.

**Data S4.** Set of r-functions used by Data S1 and S3.

**Data S5.** Zip file of R package ReadImages.

**Figure S1.** Example image 1 with porpoise sighting.

**Figure S2.** Example image 2 with porpoise sighting.

**Table S1.** Coordinates of used reference points.

**Table S2.** Tidal data of Den Helder.
