# Supplementary material for: Estimating the spatial position of marine mammals based on digital camera recordings
Source: Ecol Evol. 2015 Jan 8;5(3):578–89. doi: 10.1002/ece3.1353 (PMC4328763; doi:10.1002/ece3.1353)
Supplement: Supplementary file 1 [file ece30005-0578-sd1.docx]

## Appendix S1: Correcting for picture frame rotation

Aligning the camera perfectly horizontal may be impractical, especially when the camera is moved during operation, for instance while performing 'focal-follows' of individual porpoises. This could be corrected for by using other horizontal or vertical features (e.g. buildings) present on the recordings. However when two reference points are defined, these can also be used to correct for camera misalignment. The objective of this appendix is to illustrate how to derive the slope of an artificial horizontal line in the picture frame through *B* (*mBC*).

If the camera is aligned horizontally, a horizontal line through reference point *B* can be constructed to compute the vertical angle between the porpoise and *B*, in order to determine the distance and interior spherical angle of the sighting. In this case there is no need to correct for the rotation of the frame and the slope of the line through *B* is $m_{BC}=0$. If the two reference points *A* and *B* (both located at the sea surface) are at equal distance from the observer, the vertical angles between the centre of the Earth, the observer and the reference points (*εA* and *εB*, eq. 7) are identical (i.e. $\varepsilon_{A}=\varepsilon_{B}$), and so the horizontal line through *B* will also go through *A*. Now consider the situation where reference point *A* is further away from the observer than reference point *B* (*DOA* > *DOB*). This means that $\varepsilon_{A}>\varepsilon_{B}$, and the difference between the vertical angles ($\varepsilon_{A}-\varepsilon_{B}=\theta_{AB}$) will be positive. In the frame, *A* will now be located above the horizontal line through *B* (Fig. A1).

To correct for the rotation of the frame we need to introduce a new point *C*, which is located on the intersection between the horizontal line through *B*, and a perpendicular line through *A*.

Known variables are the coordinates of *A* and *B* in the frame and the difference between the vertical angles ($\varepsilon_{A}-\varepsilon_{B}=\theta_{AB}$), therefore the perpendicular vertical distance (in pixels) between the horizontal line through *B* (*LAC*) and reference point *A* is

$L_{AC}=\frac{\theta_{AB}}{q}$ eq. A1


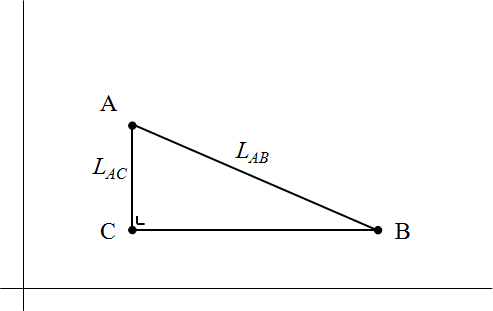


Figure A1 Reference point *A* and *B* in a scenario where the camera is perfectly horizontal and *A* is further away from the camera than *B*. *LAB* is the distance (in pixels) between *A* and *B* (eq. 5), *LAC* is the shortest distance (in pixels) between *A* and the horizontal line through *B* (eq. A1)

The slope of line *AB* is defined as

$m_{AB}=\tan\gamma=\tan(-\beta_{2})$ eq.A2

Similarly, the slope of line *BC* is given as

$m_{BC}=\tan\gamma_{2}=\tan(\gamma+\beta)=\tan(-\beta_{2}+\beta)$ eq. A3


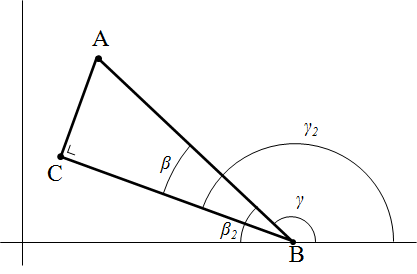


Figure A2 Reference point *A* and *B* in a scenario where the camera is tilted and *A* is further away from the camera than *B*

Now consider the situation where reference point *A* is closer to the observer, such that $\theta_{AB}$ is negative (i.e. $\varepsilon_{A}<\varepsilon_{B}$). The slope of line *BC* is now given as

$m_{BC}=\tan\gamma_{2}=\tan(\gamma-\beta)=\tan(-\beta_{2}-\beta)$ eq. A4


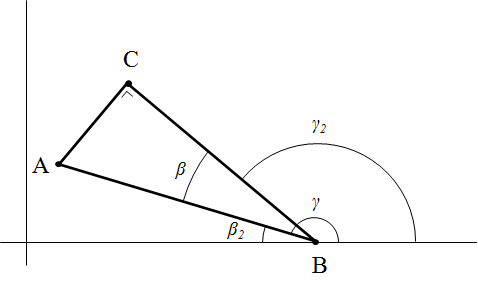


Figure A2 Reference point *A* and *B* in a scenario where the camera is tilted and *A* is closer to the camera than *B*

The apparent discrepancy between eq. A3 and A4 arises from the definition of the slope *mBC*, which can be overcome by a closer examination of *β* and *β2*. *β* can be defined as

$\beta=\sin^{-1}\left(\frac{L_{AC}}{L_{AB}}\right)$ eq. A5

*LAC* will be positive when reference point *A* is further away from the observer than reference point *B* (*DOA* > *DOB* and *θAB* > 0). When *DOA* < *DOB*, both *θAB* and *LAC* will be negative. This means that *β* will be positive when *DOA* > *DOB* (as described in the first situation above) and *β* will be negative when *DOA* < *DOB* (as described in the second situation). *β2* is described by

$\beta_{2}=\tan^{-1}\left(\frac{A_{y}-B_{y}}{A_{x}-B_{x}}\right)$ eq. A6

where *Ay*­, *Ax*, *By* and *Bx* are the pixel values of *A* and *B* respectively along the y and x-axis of the frame. *β2* will be negative when *Ay* > *By* and *Ax* < *Bx*. Because *β* and *β2* can take positive or negative values depending on both the relative position of *A* and *B* in the picture frame, and the distance from the observer, the slope of *BC* ($m_{BC}$) can now be rewritten into a single equation

$m_{BC}=\tan(\beta_{2}+\beta)$ eq. A7

## Appendix S2: Estimating the spatial position based on the horizon and a single reference point

The main section of the paper describes how to estimate the spatial position of a surfacing marine mammal using two characteristic landmarks. These reference points were used to (1) determine the individual pixel size in radians, (2) correct for the horizontal alignment of the camera, (3) determine the distance (or interior spherical angle) of the sighting, and to (4) determine the bearing of the sighting.
Below we describe how to calculate these different parameters when it is not possible to use two natural landmarks as reference points. Instead the natural horizon in combination with a single existing reference point (e.g. windmill, rock) or artificial reference points (e.g. poles) placed directly in front of the camera can used to calculate the position of the marine mammal.

*1) individual pixel size*If only a single reference point is available, the individual pixel size in radians should be determined separately before or after making the actual recordings. This can be done in different ways, for instance by following eq. 1 to 6, or by making recordings of an object with known size at a known distance. It is important to make all following recordings at the same (fixed) focal length.

*2) alignment of camera*Although in theory the camera can be perfectly horizontally aligned in the field, this may prove impractical, especially when the camera is moved around, for instance while performing 'focal-follows' on a single individuals. When no other shoreline is visible, the natural horizon can be used to correct for a tilted camera. While processing the images, two points on the horizon need to be selected (*A* and *B*, with pixel coordinates *AxAy* and *BxBy* respectively). The slope of the line between these two points is given by

$m_{AB}=\frac{A_{y}-B_{y}}{A_{x}-B_{x}}$ eq. B1

which is used to determine the intercept of the horizontal line (similar as *cBC* in eq. 8)

$c_{AB}=B_{y}-m_{AB}B_{x}$ eq. B2

*3) distance of sighting*To determine the distance to the observed marine mammal, first the interior spherical angle (*σOP*) between the observed marine mammal and the observer (eq.9 - eq.17) is calculated. Multiplied by the Earth's radius, it gives the distance from the observer to the marine mammal across the Earth's surface. In eq. 14, we use the vertical angle (εB) between the center of the Earth (*E*), the observer (*O*) and a reference point to calculate the vertical angle (εP) between *E*, *O* and the porpoise. Here, *εB* is replaced with the vertical angle (*εh*) between the horizon, the observer (*O*) and the center of the Earth:

$\varepsilon_{h}=\sin^{-1}\left(\frac{R_{E}}{R_{E}+h}\right)$ eq. B3

*4) bearing of the sighting*

When no natural reference points are available, the bearing could be determined by placing artificial reference points in front of the camera, visible on the recordings. Similar to the method presented earlier, the exact geographic location of the reference points is required, preferably determined using a DGPS. It is important to notice that errors in the determination of the exact geographical location of reference points will result in larger errors in the location estimates of surfacing marine mammals when the reference points are located in front of the camera. To estimate the bearing of a sighting, the horizontal angle between the porpoise and the reference point (*γPB*, eq. 13b) is determined. This was done by projecting the porpoise on to the horizontal line through B (eq. 8-12). However, the (artificial) reference point *Q* is not necessarily located on the horizon. Therefore, *Q* is first projected onto the horizon (eq. 9-12a, fig B1), and the distance (in pixels) between the projected point *Q’* and *P’* is used to calculate the horizontal angle (*γPB*).


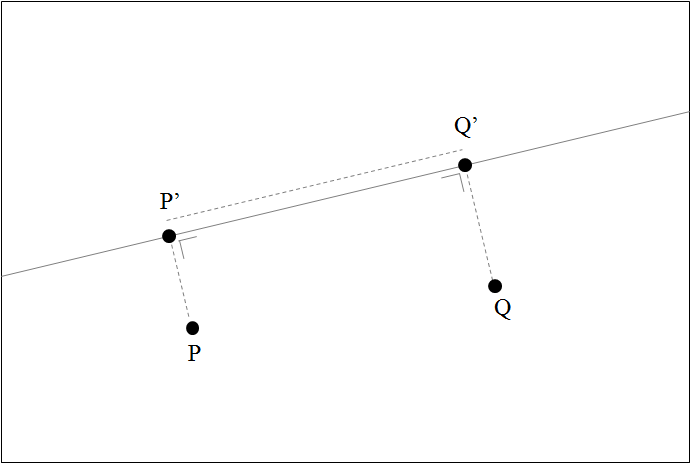


Figure B1 Schematic representation of the horizon in a tilted frame, where *P* is the porpoise, *P'* is the porpoise projected on the horizon, *Q* is the reference point and *Q'* is the reference point projected on the horizon
