# Supplementary material for: Estimating the spatial position of marine mammals based on digital camera recordings
Source: Ecol Evol. 2015 Jan 8;5(3):578–89. doi: 10.1002/ece3.1353 (PMC4328763; doi:10.1002/ece3.1353)
Supplement: Supplementary file 2 [file ece30005-0578-sd2.docx]

**Calculating harbour porpoise location in the Marsdiep with R**

*Before starting R*

- Cut frames out of de fragments by using imagegrab 4.1.3. Make sure it’s the correct versions, since older versions might show small errors in the time display. Other programs can also be used, but the filename of the frames need to be in the same format as the example frames.
- For each frame, manually note the essentials in the sightings table.
- In the folder ‘ R’ is a file ‘ frames_tot.csv’, you need to paste the times of each picture in this file (simply copy paste from the sighting form). You also need to fill in the start time of the movie, this can be found in the description of the movie file by taking the time created minus video length.
- Make sure the pictures and tables are moved to the correct hard disk and folder.
- Open code 'positions_Marsdiep_2013_01_29r' in notepad. Find ‘setwd’ and change the directory to the correct corresponding location (this has to be done in two places of the code).

*Start R 'R x64 2.15.2'*

- Copy code into R.
- Wait until the first photo appears.
- Select first reference: e.g. 25 (first click on the '2' then on '5')
- Click 'stop' to stop locator.
- Zoom in on reference: first click bottom left of target area, next top right.


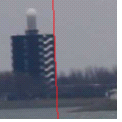


- Click on two points on the side of the building (first top, next bottom).
- Zoom in on intersection of plotted line and waterline (first click bottom left, next top right).
- Click on intersection plotted line and waterline.
- Select next reference: e.g. 23.
- Click 'stop' to stop locator.
- Zoom in on reference: first click bottom left of target area, next top right.


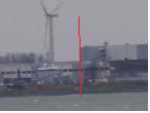


- Click on two point on the side of the building (first top, next bottom).
- Zoom in on intersection of plotted line and waterline (first click bottom left, next top right).
- Click on intersection of plotted line and waterline.
- Zoom in on porpoise (first click bottom left, next top right).
- Click on porpoise (waterline under tip of dorsal fin).
- Wait until the next photo appears.
- Repeat steps 4-17 (and use references 22 and 20).


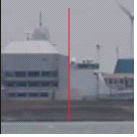

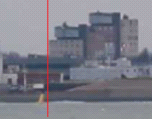


- Finally R will make the necessary calculations, wait until R is finished. During this process, all images will be displayed and the selected reference points and porpoises can be checked visually. You can find the results in the file FINAL.csv.

**But, what if..**

- I select the wrong reference point, click on the wrong place etc.?

*If you make a mistake in one picture, you can simply close R. You run R again and R will ‘remember’ were you were and proceed. This way you can redo your last picture. (Once you click the porpoise R will proceed to the next picture)*

- R starts calculations before you reach the final picture of the map?

*You probably made a mistake in copying the correct time of the picture to frames_tot.csv. Check if you made a mistake, correct it and proceed with the unprocessed pictures.*
