# Supplementary material for: Estimating the spatial position of marine mammals based on digital camera recordings
Source: Ecol Evol. 2015 Jan 8;5(3):578–89. doi: 10.1002/ece3.1353 (PMC4328763; doi:10.1002/ece3.1353)
Supplement: Supplementary file 6 [file ece30005-0578-sd6.zip › 05-01/ReadImages/html/00Index.html]

R: Image Reading Module for R

# Image Reading Module for R


---

## Documentation for package ‘ReadImages’ version 0.1.3.1

- DESCRIPTION file.

## Help Pages

|  |  |
| --- | --- |
| ReadImages-package | Utilities to read in various image formats into R. |
| clipping | Clipping image |
| imagematrix | Generate an imagematrix, i.e. primary data structure of rimage |
| imageType | Get information on color type of imagematrix |
| logo | R logo imagematrix |
| normalize | Normalization for vector and matrix |
| plot.imagematrix | Plotting an imagematrix object |
| print.imagematrix | Print information on a given imagematrix object |
| read.jpeg | Read JPEG file |
| ReadImages | Utilities to read in various image formats into R. |
| rgb2grey | Convert color imagematrix to grey imagematrix |
