# Supplementary figures and images for: Estimating the spatial position of marine mammals based on digital camera recordings
Source: Ecol Evol. 2015 Jan 8;5(3):578–89. doi: 10.1002/ece3.1353 (PMC4328763; doi:10.1002/ece3.1353)

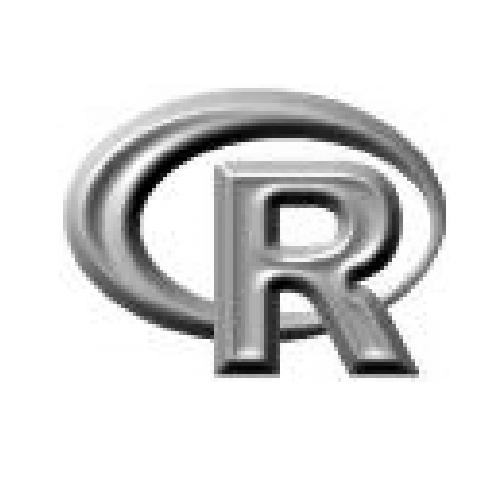

Supplement: Supplementary file 6 [file ece30005-0578-sd6.zip › 05-01/ReadImages/data/Rlogo.jpg]

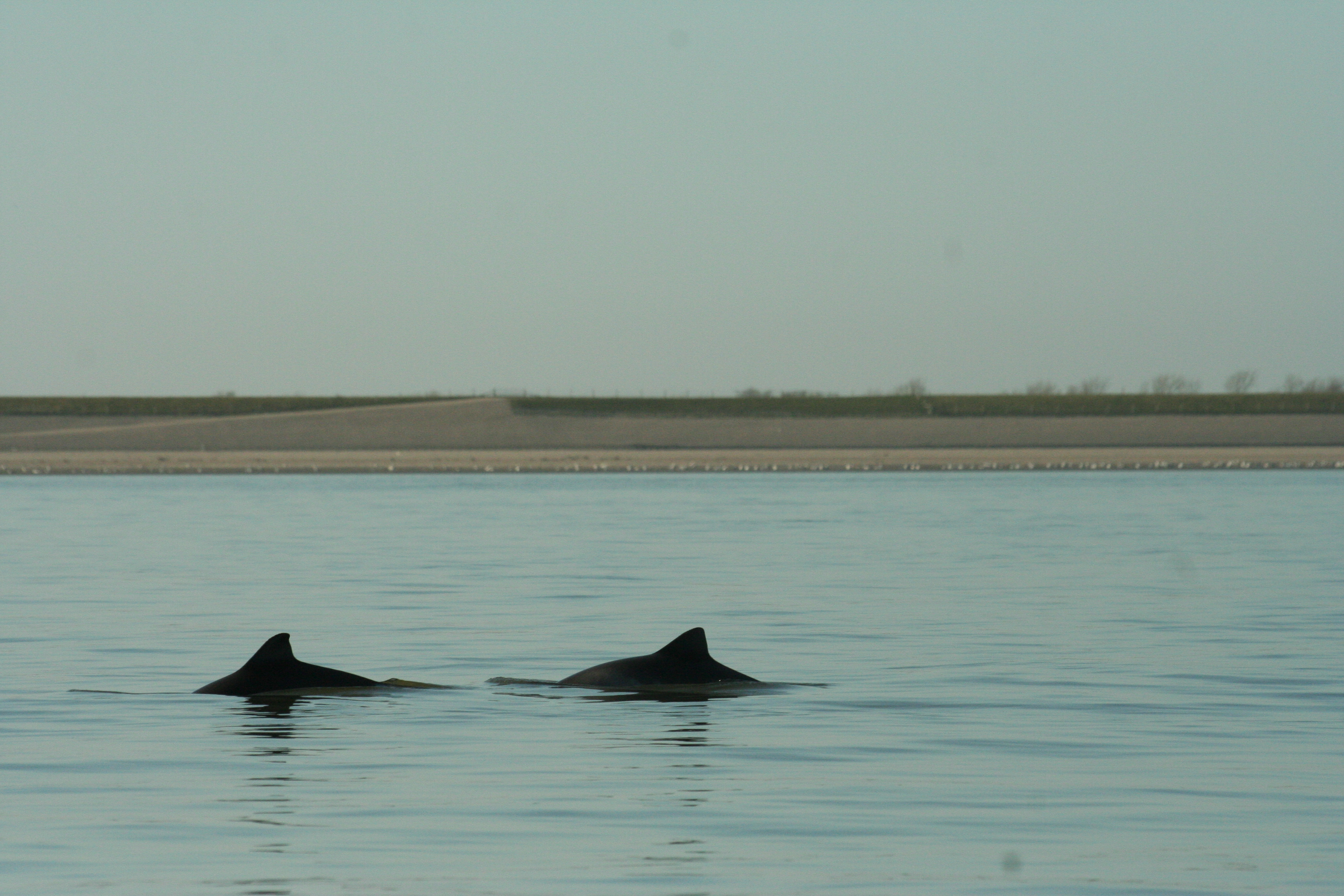

Supplement: Supplementary file 7 [file ece30005-0578-sd7.jpg]

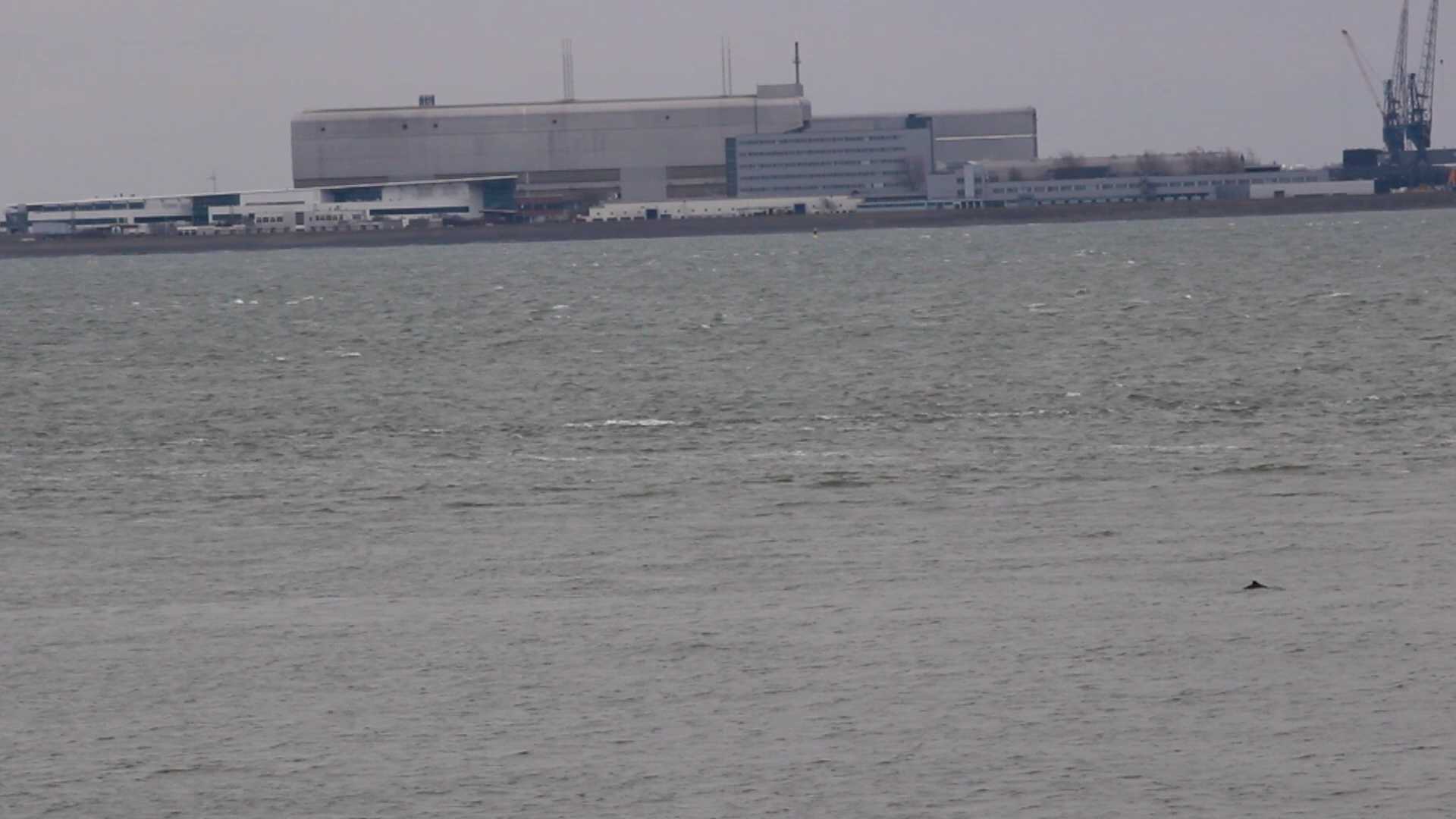

Supplement: Supplementary file 8 [file ece30005-0578-sd8.jpg]
